# Microneedles: A New Frontier in Nanomedicine Delivery

**DOI:** 10.1007/s11095-016-1885-5

**Published:** 2016-02-23

**Authors:** Eneko Larrañeta, Maelíosa T. C. McCrudden, Aaron J. Courtenay, Ryan F. Donnelly

**Affiliations:** School of Pharmacy, Queen’s University Belfast, 97 Lisburn Road, Belfast, BT9 7BL UK

**Keywords:** drug delivery, microneedles, microparticles, nanomedicine, nanoparticles, vaccines

## Abstract

This review aims to concisely chart the development of two individual research fields, namely nanomedicines, with specific emphasis on nanoparticles (NP) and microparticles (MP), and microneedle (MN) technologies, which have, in the recent past, been exploited in combinatorial approaches for the efficient delivery of a variety of medicinal agents across the skin. This is an emerging and exciting area of pharmaceutical sciences research within the remit of transdermal drug delivery and as such will undoubtedly continue to grow with the emergence of new formulation and fabrication methodologies for particles and MN. Firstly, the fundamental aspects of skin architecture and structure are outlined, with particular reference to their influence on NP and MP penetration. Following on from this, a variety of different particles are described, as are the diverse range of MN modalities currently under development. The review concludes by highlighting some of the novel delivery systems which have been described in the literature exploiting these two approaches and directs the reader towards emerging uses for nanomedicines in combination with MN.

## INTRODUCTION

**Fig. 1:**
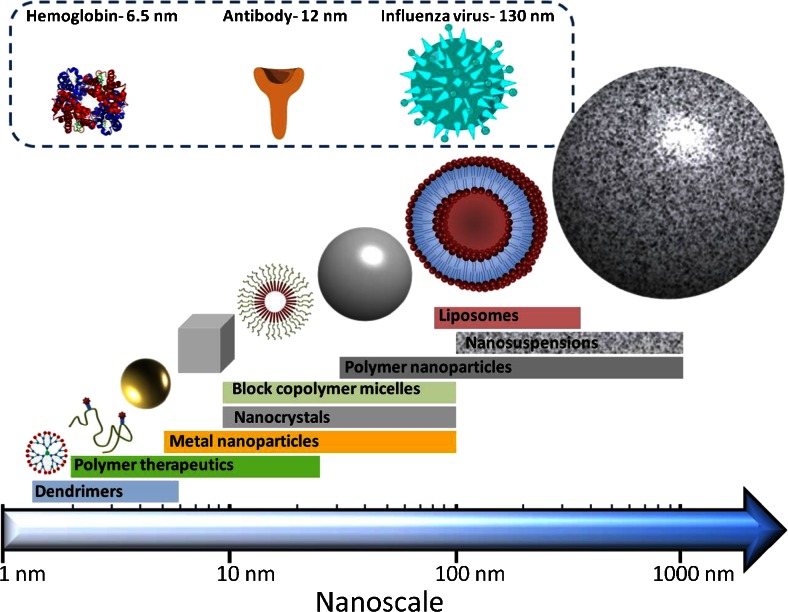
Structures of different nanomedicines and their approximate sizes. For comparative purposes, the sizes of biological nanostructures are shown at the top of the figure. Reproduced with permission from the British Society For Nanomedicine.

The interest on nanotechnology applied to healthcare (also called nanomedicine) have suffered an exponential growth during the last 25 years. There are different definitions of the term “nanomedicine” but generally it can be defined as the use of nanoscale or nanostructured materials in medicine with unique medical effects related with their structure (1). Figure 1 shows some examples of different nanomedicines and their approximate sizes. The applications of nanomedicine cover treatment, diagnosis, monitoring, and control of biological systems (2). Consequently, drug delivery is one of the applications covered by nanomedicine. Nanoparticles (NPs) have been extensively used to deliver conventional drugs, recombinant proteins, vaccines and nucleotides (3). According to the National Nanotechnology Initiative (American National Standards Institute), NPs are particles with all dimensions between 1 and 100 nm (4). However, in the scientific literature the term “nanoparticle” can be found in a wide variety of works referring to particles larger than 100 nm. NP formulations show unique size-dependent, physico-chemical properties and can be made of a wide variety of compounds such as lipids, sugars, degradable or non-degradable polymers, metals and organic or inorganic compounds (4). NPs exhibit numerous advantages over traditional drug delivery systems. Nanoparticulate systems allow sustained drug release for a prolonged period of time and additionally provide protection for encapsulated materials against chemical or proteolytic degradation (4). Besides, they can be modified with certain ligands in order to target them to certain parts of the body (5). NPs formulations can be administered *via* different routes such as oral, intravenous, pulmonary, nasal and ocular (6). Oral administration continues to be the preferred route of administration despite the fact that it presents various drawbacks such as the potential degradation of the drug in the gastrointestinal tract. Additionally, many drugs can suffer first-pass effects, resulting in reduction of drug concentration before it reaches the systemic circulation (7,8). NPs are promising carriers as they can protect the medicine from degradation and/or interact with the mucosal surface and therefore increasing their bioavailability (6,9,10). On the other hand, parenteral route do not present all the limitations associated with the gastrointestinal track and allows a quick onset of action after administration and a reduced dosage of the drug (11). Also, parenteral route allows the administration of medicines directly into the bloodstream or into a specific tissue. This route suffers from poor patient compliance as it is often associated with pain due to the use of hypodermic needles. In addition, the use of needles and syringes generates sharps medical waste thus eliciting the risk of disease transmission by needle re-use, especially in developing countries (12). An effective alternative to parenteral and oral delivery systems is the use of transdermal delivery systems. They are non-invasive and can be easily self-administered. The primary barrier to transdermal delivery is the *stratum corneum* (*SC*)*,* the outermost layer of the skin, a lipophilic barrier that limits the number of drugs capable of being administered *via* this route (13,14). Suitable drugs should be of moderately low molecular weight, have octanol-water partition coefficients that heavily favour lipids and also exhibit reasonable potency (low dose) (15–18). To this end, transdermal administration of macromolecules, peptides or hydrophilic drugs has, to date, remained relatively unexploited. Despite all of these limitations, several medicinal preparations have been commercialised since 1979. In fact, in 1979, the first transdermal product was approved for use in the United States. Currently, the transdermal route vies with oral administration as the most effective novel research area in active pharmaceutical ingredient (API) delivery. The wealth of the worldwide transdermal patch market is approaching $32 billion (American), yet is based on only 20 drugs (19). Therefore a wide variety of novel systems have been evaluated to try to enhance the permeability of drugs through the *SC* (8), including the use of NP-based formulations (4,20). Despite all these promising features however nanoparticulate systems can achieve only minimal permeation through the *SC* (20).

A further novel strategy to overcome the *SC* is the use of microneedles (MN). MN are minimally invasive devices that by-pass the *SC* barrier and thus achieve transdermal drug delivery (7,21–23). These devices have garnered much attention in the pharmaceutical field, particularly over the past decade due to their ability to facilitate the administration of drugs and vaccines across the skin but also based on their novel use in the extraction of biological fluids for monitoring purposes (24,25).

The development and exploitation of combinatorial approaches to capitalise on the advantages of both NP and MN delivery systems have increased steadily over the past 15 years. This review aims to comprehensibly chart the development of such MN/NP combinatorial applications.

## SKIN STRUCTURE AND BARRIER PROPERTIES WHICH INFLUENCE NANOPARTICLE AND MICROPARTICLE PENETRATION

**Fig. 2:**
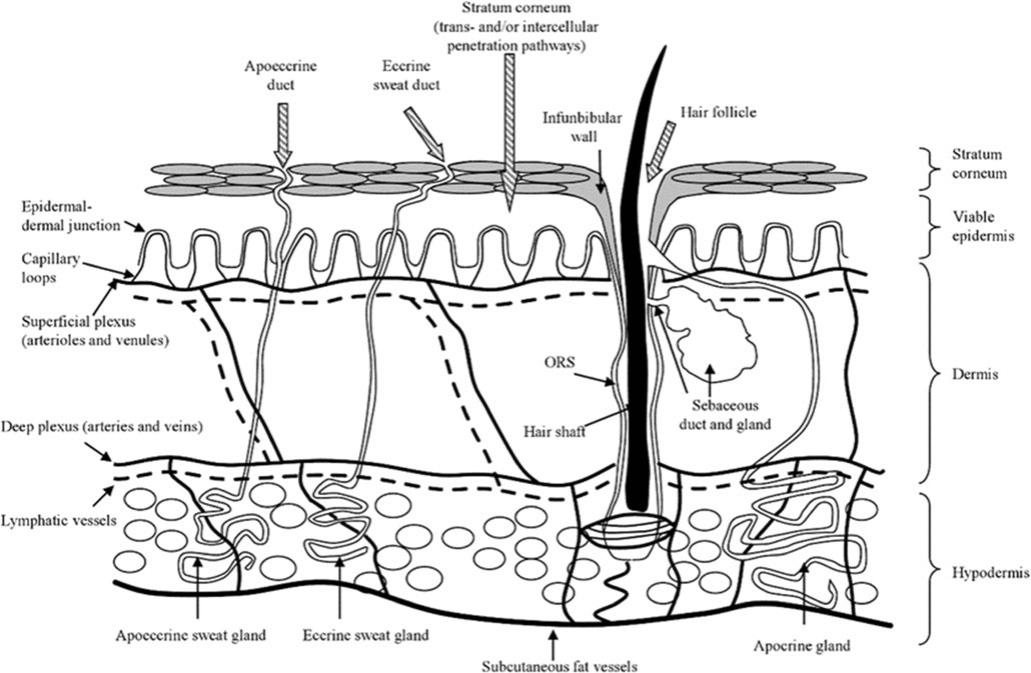
Diagrammatic representation of skin structure. Reproduced with permission from (27).

The skin is the largest and one of the most complex organs in the human body and carries out a wide range of functions (26). This organ contains and protects the internal body organs and fluids, providing temperature and, to some extent, humidity control for the body. Moreover, it acts as a heat, cold, touch and pressure sensor organ connecting with the central nervous system. As can be seen in Fig. 2, the multi-layered nature of human skin can be resolved into three distinct layers: the epidermis, beneath which lies the much larger dermis and, finally, the deepest layer, the subcutis, also termed the hypodermis (28).

The outermost layer of the epidermis, and thus the skin, is the *SC*. It is now well accepted and documented, that this layer constitutes the principal barrier for penetration of most drugs and nanomedicines (NMs) (20,29). The *SC* typically has a thickness of 10 μm but factors such as the degree of hydration and the location of the skin influence it’s thickness. For instance, the *SC* on the palms and soles can be between 400 and 600 μm in thickness (29), whilst hydration can lead to a 4-fold increase in thickness (30). The *SC*is formed mainly by 10–25 rows of dead keratinocytes, now called corneocytes, embedded in the secreted lipids from lamellar bodies (29). The bricks and mortar model is a common representation of this layer (30). Corneocytes are polygonal, elongated and flat, thus resembling ‘bricks’ (18). On the other hand, the ‘mortar’ is formed by a continuous interstitial lipid matrix. These lipids are arranged in the lamellar phase (alternating layers of water and lipid bilayers), with some of the lipid bilayers exhibiting a certain degree of crystallinity (31). The multiple lipid bilayers residing in the intercellular space are thought to be responsible for the barrier properties of the *SC*. These bilayers prevent desiccation of the underlying tissues by inhibiting water loss and limit the penetration of substances from the external environment (31). The *SC* also has hydrophilic regions that form aqueous pores facilitating the trans-epidermal polar route of skin absorption (19). Passive diffusion is the main mechanism of transport of substances through this layer. The three acknowledged routes of passive diffusion are: the transcellular, the intracellular and the appendageal routes (19). The transcellular route involves diffusion through the lipid matrix occupying the intercellular spaces of the keratinocytes while the transcellular route involves permeation through the keratinocytes (19). Depending on the chemical nature of the substance which is diffusing through the skin, penetration *via* the intercellular pathway could involve polar or lipidic routes. Finally, the appendageal route involves permeation through hair follicles, sebaceous glands and sweat glands. Typically, the intracellular route can be used by agents with sizes ranging between 5 and 36 nm while larger molecules (10–210 μm) may penetrate the skin through the trans-follicular route (20). The mode of permeation of a NP through the skin cannot be generalised however as many factors affect NP permeation characteristics. For example, the surface of the skin has an acidic pH (4.2 to 5.6) that facilitates antimicrobial defence and restricts inflammation, among other functions (19). This acidic nature can hinder NP permeation as it can alter the physico-chemical properties of the particle. One such example are zinc oxide NPs as acidic pH affects the aggregation and dissolution kinetics of these particles and consequently affects its permeability through the skin (32).

In addition to the *SC*, the viable epidermis is another important barrier for NP penetration. The epidermis is a stratified epithelium that lies directly above the dermo-epidermal junction. In contrast to the *SC*, the epidermis has a hydrophilic nature that limits the permeation of lipophilic agents. The presence of proteolytic enzymes that can degrade foreign substances, in combination with the presence of tight junctions within the skin layers, limits the permeation of nanomedicines (NMs) through the viable epidermis (19).

Skin appendages, such as sweat glands and pilosebaceous units, on the surface of the skin facilitate the penetration of different agents, including NMs into the skin. Sweat glands are coiled tubular glands that are extended from the dermis to the *SC* (2–5 mm length). Their main functions are thermoregulation and excretion of bodily waste. Pilosebaceous units consist of hair follicles with one hair (hair infundibulum). These follicles have sebaceous glands associated. Despite the fact that these units provide large openings on the skin surface, their density is low and therefore their utility in NP delivery is limited (19).

## CONVENTIONAL NANOPARTICLES AND MICROPARTICLES FOR TRANSDERMAL DRUG AND VACCINE DELIVERY

The term “conventional” in this article refers to systems based on passive permeation through the *SC,* rather than the disruption of the same. Additionally, the term “nanoparticle” will be used for particles with sizes smaller than 1000 nm.

**Fig. 3:**
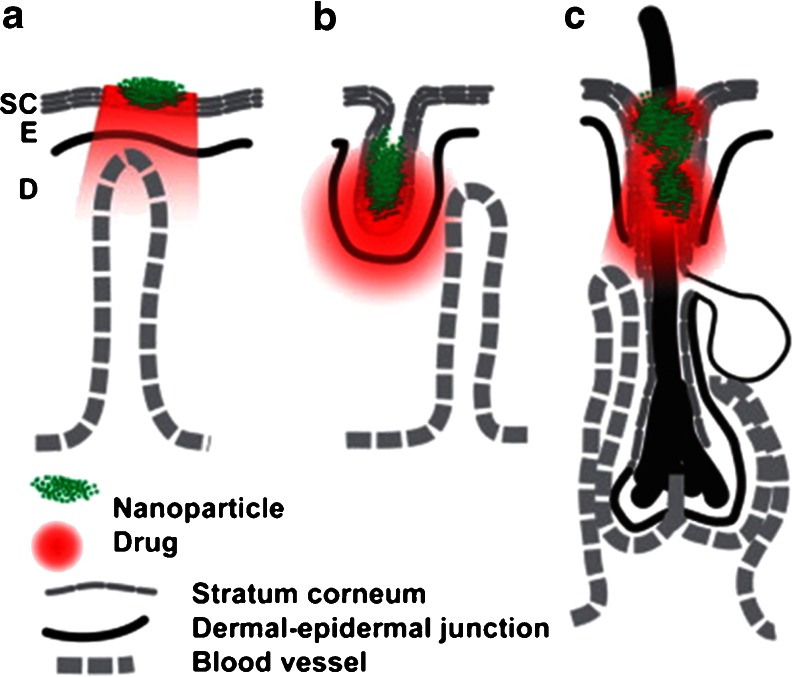
Sites in skin for NP delivery. Topical NP drug delivery takes place in three major sites: *SC* surface (**a**), furrows (**b**), and openings of hair follicles (**c**). The NPs are depicted in green and the drug in red. Other sites for delivery are the viable epidermis (**e**) and dermis (**d**). Reproduced with permission from (4).

Transdermal NP delivery has, to date, been used to elicit local effects (4). Nevertheless, the exact mechanism of NP penetration remains unclear and there is an ongoing debate surrounding this mechanism (4,20). The potential skin sites for targeting NPs are the *SC*, furrows and hair follicles (Fig. 3) (4). Thus far, NP-mediated drug delivery into deeper layers of the skin (epidermis and dermis) without breaching the *SC* has met with limited success (4,20,33,34).

### Lipidic Vesicles

Liposomes, niosomes®, ethosomes®, transfersomes®, proliposomes®, pharmacosomes® and vesosomes® are all different types of lipidic vesicles. All these vehicles normally present particle sizes larger than 100 nm and a certain degree of deformability (20). Liposomes are the most popular nanocarriers (34). They are formed by vesicles that enclose an aqueous environment by one or multiple lipid bilayers (mainly phospholipids and/or cholesterol) (35). Niosomes® were developed to improve the stability of liposomes by including non-ionic surfactants in their structure (20). Ethosomes® are similar to liposomes but can lead to enhanced skin penetration due to the fact that they represent a more elastic, lipidic vesicle (35). Transferosomes® are ultra-deformable vesicles made of a mixture of phospholipids supplemented with surfactants (35). Proliposomes® are free flowing particles made of drugs, phospholipids and a water-soluble porous powder working as a support that increases the surface of dry lipid (36). Pharmacosomes® are colloidal dispersions of drugs covalently linked to phospholipids (36). Finally, vesosomes® are vesicles inside another vesicle designed mainly for transcutaneous immunization (36). Transdermal delivery of drugs using these vehicle types has been extensively studied over the course of the last 35 years. These nanocarriers have been extensively used for the topical delivery of anaesthetics (37,38) or anti-inflammatory (39–41) compounds. Liposomes have been used to deliver antifungal, antibiotics and a wide variety of peptides to skin tissues by topical application (36). Furthermore, liposomes have been shown to have promising properties for the treatment of skin cancer *via* the delivery of prodrugs, such as 5 aminolevulinic acid, for photodynamic therapy or by delivering DNA repair enzymes to patients with skin cancer (36). Despite these topical uses, liposomes have little or no value as carriers for transdermal drug delivery as these vesicles are not able to cross the SC (36). In contrast, niosomes®, ethosomes® and transfersomes® hold more potential in the field of transdermal delivery as they allow deeper penetration into the skin than conventional liposomes.

### Lipid Nanoparticles

Lipid nanoparticles include solid lipid nanoparticles (SLN) and nanostructured lipid carriers (NLC). These nanocarriers were developed to overcome stability problems associated with liposomes (42). SLN are composed of a single lipid that is solid at body temperature, coated with a surfactant acting as stabiliser (43). Conversely, NLC are formed by a solid lipidic matrix comprising a mixture of liquid and solid lipids encapsulating a liquid lipidic nanocompartment (43). SLN and NLC are rigid and can possess either positive or negative charge, with average sizes ranging between 50 and 1000 nm (20). SLN and NLC have been used mainly for cosmetic and dermatological applications such as the treatment of eczema and acne (20,42,43).

Worth noting is that fact that the enhanced drug permeation mechanism exhibited by these nanocarriers has been suggested to be due to the formation of an occlusive film on the surface of the skin rather than due to the permeation of the particles through the *SC* (20).

### Polymeric Nanoparticles and Microparticles

The category of polymeric nanoparticles and microparticles may include polymeric micro/nanospheres and micro/nanocapsules. The former are polymeric particle matrices while the latter are vesicles (generally oily) covered with polymers. Both types of particles can be produced in a broad range of sizes possessing either positive or negative charges (20). These vehicles have been used extensively as drug delivery systems in a wide variety of different locations in the body (20). These rigid nanoparticulate systems do not penetrate the *SC* but have been shown to penetrate into hair follicles (20,44–46). Polymeric NPs have been extensively used as topical delivery systems (47). Natural polymers (e.g. chitosan, alginate, gelatin and albumin) have been extensively used to prepare NPs for topical delivery. Among many natural polymers, chitosan has been frequently utilised in the preparation of NPs for topical skin delivery (47). It has been used for the topical delivery of retinol, aciclovir, quercetin, and even macromolecules such as antisense oligonucleotides and plasmid DNA (47–49). In addition to NPs made using natural polymers, there are a wide variety of studies using synthetic polymers for the preparation of topical NP delivery systems. These synthetic polymers can be structured into two different groups: biodegradables and non-biodegradables. Poly(d,l-lactic-co-glycolic acid) (PLGA) is the most commonly used biodegradable polymer for the preparation of NPs. These NPs have been used for the delivery of indomethacin, spantide II and ketoprofen, in addition to other therapeutic agents (50,51). Finally, NPs formulated with non-degradable polymers, such as polyacrylates, have also been studied for cutaneous delivery of active compounds but to a lesser extent (47).

### Microemulsions

Microemulsions include formulations composed of stable emulsions of aqueous and oily phases in the presence of a surfactant or cosurfactant (52). Droplets formed exhibit high flexibility with sizes generally less than 150 nm (20). Microemulsions have been used extensively to enhance the permeation of drugs (52) and even larger molecules such as plasmid DNA (53). Intact droplets have not been shown to penetrate into the skin (20) but rather particles collide with skin structures and then release their cargos (20). Microemulsions have been extensively used for cutaneous delivery as this nanocarrier displays more pronounced retention in the skin layers, rather than acting *via* percutaneous permeation (46). Microemulsions have been used for both cosmetic (i.e. delivery of ascorbic acid and lycopene) (54,55) and therapeutic applications (46) in the skin. Therapeutic applications include the delivery of a wide variety of molecules such as anesthetics, cyclosporine, lidocaine, alpha-tocopherol, temozolomide hexyl ester, ascorbyl palmitate, desmopressin acetate andpaclitaxel (46). Despite their limited efficacy in transdermal delivery however, microemulsions have been used successfully for transdermal delivery of several molecules, including testosterone, nicotinic acid, lidocaine, estradiol and sodium diclofenac (46).

### Metallic and Mineral Nanoparticles

Metallic and mineral nanoparticles include magnetic NPs, quantum dots (QD), titanium and zinc oxide NPs, carbon nanotubes and fullerenes. Magnetic NPs are iron derivative particles with sizes ranging between 2 and 100 nm (20). The applications of this type of particle include: imaging for the diagnosis of skin diseases, magnetic assisted drug delivery and drug targeting, hyperthermia treatment of tumours and magnetic transfection of cells (19,20). Baroli *et al.* demonstrated that when administered topically, this type of particle was found to permeate into the deeper layers of the *SC*, hair follicles, SC-*stratum granulosum* interface and in some rare cases in the viable epidermis (56).

QD are core-shell nanomaterials formed by group IV-VI elements, or group IV elements alone of the periodic table (57). They have been used extensively for biomedical research purposes as they possess unique spectral and optoelectronic properties (57). They are often coated with different surface coatings, depending on their intended applications. The use of QD in skin permeation studies have been investigated by various different research teams (58–62). Despite this however, there are, to date, no definitive conclusions about the mechanism of permeation of QD through the skin. Several authors reported QD permeation through porcine skin and murine skin (60,62) while other authors reported that no conclusive QD penetration was observed (59,61). Worth noting is the fact that none of these publications dealtwith QD permeation through human skin and as such, all of these findings should be evaluated carefully before extracting any definitive conclusions.

Titanium and zinc oxides are examples of nanomaterials with the ability to scatter UV light. For this reason, they are used as an integral component of sunscreens in the cosmetic industry (19). They are normally coated with aluminium oxide, silicon dioxide or silicon oils increasing their sizes and dispersion stabilities (20). Zinc oxide particle sizes fall between 30 and 200 nm, while titanium oxide particle sizes can be up to several microns (19). Nohynek *et al.* showed that both zinc and titanium oxide particles penetrate only into the outermost layers of the *SC* (63). In contrast however, Kimura *et al.* reported that zinc and titanium oxide NPs did not migrate beyond the surface of the skin into the viable epidermis and dermis (64).

Carbon nanotubes and fullerenes are carbon-based nanomaterials in either the shape of single/multi-walled hollow tubes or hollow spheres, respectively (65). Rouse *et al.* demonstrated the ability of a coupled fullerene-FITC-peptide compound (3.5 nm) to diffuse into skin by passive penetration when mechanical stress was applied to the skin (66). Carbon nanotubes have been used to enhance transdermal permeation of different compounds such as siRNA (67) and indomethacin (68). Nevertheless, Degim *et al.* reported that despite enhancing indomethacin permeation, the selected carbon nanotubes were unable to penetrate through the skin (68).

## MICRONEEDLE-MEDIATED TRANSDERMAL DELIVERY OF NANOPARTICLES AND MICROPARTICLES

### Microneedle Modalities

**Fig. 4:**
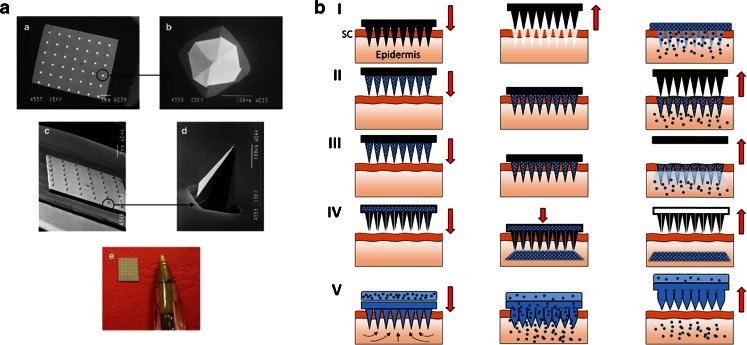
Images of a typical silicon MN arrays (**a**). Scanning electron microscopy (*SEM*) images taken of a typical silicon MN array (**a**–**d**) and a digital photograph of a typical silicon MN array (**e**). Schematic representation of the main MN modalities (**b**). Solid MNs (I); coated MNs (II); dissolving MNs (III); hollow MNs (IV) and hydrogel-forming MNs (V). Reproduced with permission from (69).

MN devices are composed of arrays of micron-size needles (Fig. 4a). The dimensions of these micro-projections generally range from lengths of only a few micrometres to those as long as 2000 μm (23). When applied to the skin surface, they bypass the *SC* without stimulating dermal nerves due to the short length of the individual needles (7,21,22). The holes created in the skin by the MNs can be used to deliver drugs from the skin surface to the dermal microcirculation (7,21,22). Therefore, MNs are considered pain-free, minimally invasive devices that combine the advantages of traditional transdermal drug-delivery systems with the targeting of conventional hypodermic needles (21). Extensive research has been carried out in the field of MN technology using different materials, MN designs and fabrication methods (7,21–23,70). In the literature, five main types of MN design have been described (23), namely solid, coated, dissolving, hollow and hydrogel-forming MNs.

Solid MNs are utilised in an approach termed, ‘poke with patch’ (7,21,22). This strategy involves a two-step process (Fig. 4b I). In the first step, solid MN arrays are applied to the skin and subsequently removed. This process creates micro channels in the skin surface onto which a conventional drug formulation is applied. Permeation of the active molecules from this formulation occurs *via* passive diffusion through the created micro channels. Solid MNs are normally prepared using silicon (71–73), metals or polymers (74). A wide variety of studies have been published describing the ability of solid MN arrays to enhance transdermal permeation of different molecules such as insulin (75), calcein (74,75), naltrexone (76) or proteins (75). Additionally, this type of MN modality has been used in combination with other strategies, such as iontophoresis, to enhance the efficacy of transdermal delivery (77).

Coated MNs consist of a solid MN array (normally made of silicon or metal) coated with a drug/vaccine formulation (7,21,22). Coated MNs are applied to the skin and after insertion the coated formulation is deposited into the skin (Fig. 4b II). This approach is denoted as ‘coat and poke’ (22). Different strategies can be employed to successfully coat the formulations onto the surface of the MNs (78–80). Coated MNs have been used to delivery vaccines, proteins, peptides and DNA into the skin (79,81–84). The use of coated MNs may prove a viable option for vaccination purposes or for the delivery of potent drugs but is not suitable for other applications as the MNs cannot be loaded with large amounts of active molecule.

Dissolving MNs are made of a soluble/biodegradable matrix that includes the active substance. Conventionally, micromolding techniques are used to produce this type of MN (7). Following insertion, the needle matrices dissolve/biodegrade in the skin, thus releasing their cargoes (Fig. 4b III) (7,21,22). Commonly these types of arrays are made of sugars, carbohydrates or synthetic polymers (7,23) and have been utilised to deliver a range of different substances including insulin (85,86), low molecular weight heparin (87), ovalbumin (88,89), adenovirus vector (89), vaccine antigens (90), photosensitizers and precursors (73,91), in addition to low molecular weight drugs (92). Dissolving MNs have also been used successfully in combination with other permeation enhancing strategies such as iontophoresis (93).

Hollow MNs, as the name suggests, consist of hollow needles that, following insertion, facilitate injection of a fluid medication into the skin (Fig. 4b IV) (7,21,22). Hollow MNs allows the delivery of molecules in a continuous fashion. These devices have been produced using different materials such as silicon (94), metal (95), hollow glass (96), polymers (97) and ceramic (98). Hollow MNs have been extensively used to deliver insulin (94,96,99).

Finally, hydrogel-forming MN arrays consist of MN made of a swelling material with a drug reservoir attached to the baseplate of the array (100–103). After insertion, the array absorbs interstitial fluid leading to the diffusion of the drug from the backing through the swollen microprojections into the skin (Fig. 4b V) (101,102). These MN arrays are produced mainly using synthetic polymers that can be easily crosslinked by chemical or physical methods (104). Hydrogel-forming MN arrays have been employed to deliver a wide range of different molecules from small molecules (91,101,102,105) to high molecular weight compounds (101,102,105).

### Microneedle-Assisted Nanoparticle/Microparticle Permeation

Different types of MNs have been used to enhance the permeability of NPs and microparticles (MPs). A wide variety of research studies can be found in the literature dealing with MN-assisted permeation of nano- and micro-metric particles. In the first instance, we will describe those studies which mainly focused on permeation optimisation of NPs and MPs when used in combination with MNs. Therapeutic applications of NPs or MPs used in combination with MNs will be described in subsequent sections.

**Fig. 5:**
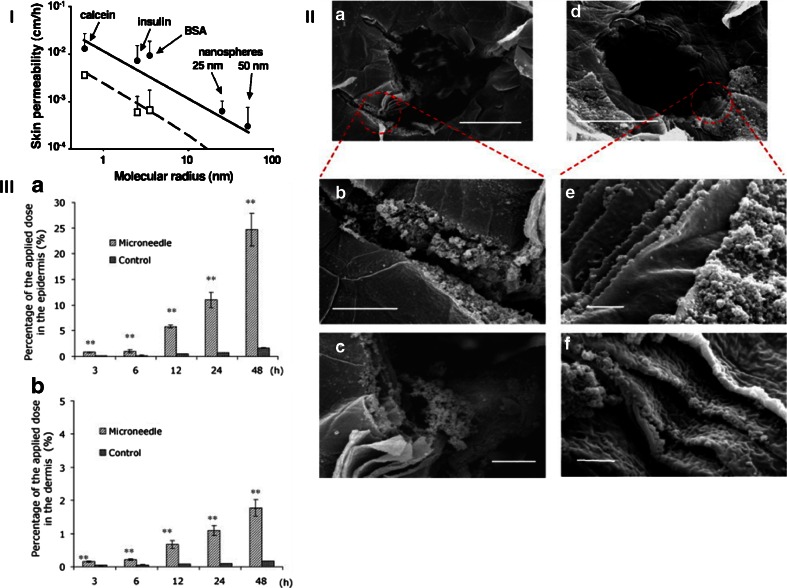
Skin permeability to molecules and particles of different sizes after treatment with MN arrays (I). The permeability of human cadaver epidermis was increased by orders of magnitude with a 400-needle array inserted (□) and after the array was removed (•) for calcein, insulin, BSA, and latex nanospheres of 25 nm and 50 nm radius. Predictions are shown for needles inserted (*dashed line*) and needles removed (*solid line*) by using a mathematical model. Scanning electron micrographs of human epidermal membranes treated with a silicon MN device and a subsequent topical application of a fluorescent nanosphere formulation (II). (**a** and **d**), scale bar = 50 μm; (**b** and **c**), scale bar = 10 μm; (**e** and **f**), scale bar = 2 μm. Percentage of the applied dose of NPs deposited within the epidermis and dermis within a 48-h period (III). The skin without MN treatment was used in the control groups. Reproduced with permission from (75,106,107).

The first report of NP permeation through the skin using MNs was published in 2003 by McAllister *et al.* (75). In this work, the ability to enhance permeation of compounds with different molecular radius (calcein, insulin, BSA and NPs) using solid MN arrays was evaluated. As a model for NPs, two types of polystyrene latex nanospheres were employed (25 and 50 nm of radius). The skin permeability of all the evaluated compounds, depicted as a function of their molecular radius is presented in Fig. 5 I. Mathematical modelling was used to predict the permeation mechanism supporting the idea that transport takes place *via* diffusion through water-filled holes in the skin. In a similar way, Coulman *et al.* studied the diffusion of fluorescent polystyrene nanospheres (100–150 nm in diameter) across skin pre-treated with silicon MN arrays (106). The results of this work are consistent with the results presented previously by McAllister *et al.* (75). Furthermore, in this work, additional factors were studied, such as surface charge of the particles. The permeation of the NPs across human epidermal membranes was tested with variable results, mainly due to the complexities that are associated with the use of multi-layered tissue structures (human epidermal membrane) for MN assisted NP delivery. As can be seen in Fig. 5 II, NPs were found on the interior surface of the created microchannels and adsorbed to the corneocyte surfaces of the disrupted epidermal membrane. Similar results were obtained by Zhang *et al.* using PLGA NPs loaded with coumarin 6, in combination with solid silicon MN arrays (107). The two main findings of this work were: (i) following administration, a larger amount of NP was found in the epidermis rather than in the dermis (Fig. 5 III) and (ii) the permeation rate of the NP was dependent on the NP concentration reaching a limit for higher concentrations (ca. 1.6 mg/mL). The main results obtained previously by others using solid MN arrays in combination with NPs/MPs formulations were corroborated by Gomaa *et al.* (108). The permeation of PLGA NPs of differing sizes containing nanoencapsulated dyes (Rhodamine B and fluorescein isothiocyanate) across excised porcine skin previously treated with polymeric MN arrays was evaluated. Effect such as the particle size, particle surface charge, NP composition or chemical nature of the encapsulated dye strongly influenced the permeation of the NPs. Following a different strategy, Zhang *et al.*, in two different studies, researched MN-assisted permeation of metallic MPs into an agarose gel that mimics the skin (109,110).

**Fig. 6:**
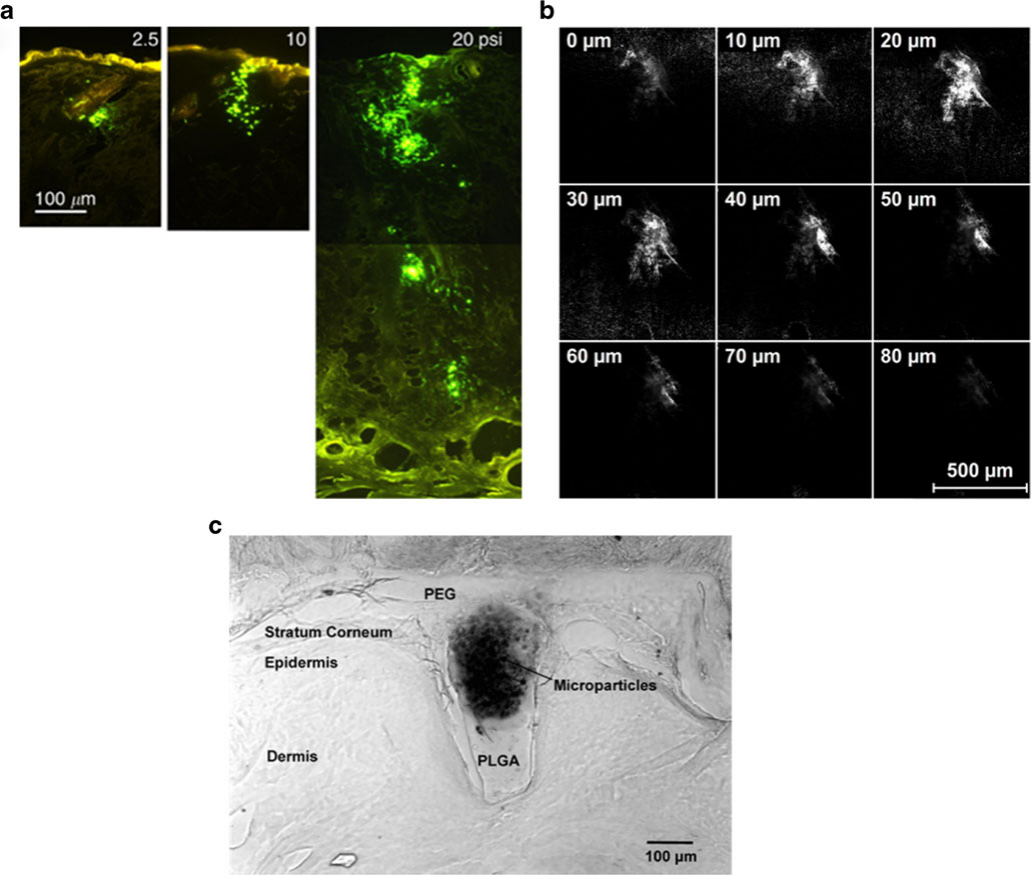
Fluorescence micrographs of histological sections after microinjection of 2.5 μm fluorescent microspheres into hairless rat skin *in vivo* under pressures ranging from 2.5 to 20 psi *via* the same needle and loading for the same time periods (**a**). Confocal microscopic images of 0.7 μm large fluorescent polystyrene microspheres at different depths in chicken tissue after injection with a MEMS syringe (**b**). Multi-layered MN inserted into pig cadaver skin, cryo-sectioned vertically and viewed by brightfield microscopy (**c**). Reproduced with permission from (96,111,112).

A different strategy to enhance NP and MP permeation across the skin is the use of hollow MNs. Following this approach Wang *et al.* (96) studied the microinjection of polymeric MPs using a single hollow glass MN. The selected MPs had sizes of 2.5 and 2.8 μm. This work evaluated the ability to use hollow MNs to inject different compounds intradermally. The particles were successfully injected into the skin of hairless rats *ex vivo* and *in vivo.* Nevertheless, in order to obtain optimal delivery, different aspects of the application process should be tailored and adjusted, such as the injection pressure enlisted or the retraction of the MN after insertion (Fig. 6a). Similarly Häfeli *et al.* developed a syringe-type device equipped with silicon hollow MNs to inject different substances into the skin (111). They tested this device by injecting an aqueous suspension containing blue polystyrene microspheres (0.93 μm in diameter) and fluorescent polystyrene microspheres (0.7 μm in diameter). Using confocal microscopy, the penetration depths of the microspheres after injection were evaluated elucidating that the particles can be detected at a depth of 70 μm but the maximum particle concentration obtained was found to be approximately 20 μm underneath the surface of the skin (Fig. 6b). The use of hollow MNs to inject NP is not restricted to the skin. Ocular drug delivery is a research area that presents interesting challenges, especially when administering drugs to the back of the eye. MN arrays in combination with MPs and NPs can play an interesting role in this niche area. Jiang *et al.* demonstrated the ability of hollow, glass MN arrays to inject NPs and MPs into the sclera in a minimally invasive manner (113). Poly(lactic acid) (PLA) NPs containing Nile Red were injected using an insertion-retraction method. The injection of latex fluorescent MPs required the addition of enzymes to the tissue to disrupt scleral structure. The same system was used by Patel *et al.* to inject NPs (size ranging from 20 to 1000 nm) inside the suprachoroidal space of the eye (114). The injection of the particles was optimized illustrating that an increase in infusion pressure, MN length and intraocular pressure facilitates the injection process. Furthermore, decreasing NP size promotes the injection process. Kim *et al.* showed recently reported of the use of hollow MNs to deliver particle-stabilized emulsion droplets to the back of the eye of rabbits *in vivo* (115). The emulsion was composed of a high-density perfluorodecalin core (≤35 μm in diameter) surrounded by fluorescein-tagged polystyrene NPs (polymeric drug carrier model). After infusion of the emulsion into the suprachoroidal space of the eye, at least 50% of the injected NPs were found to be near the macula or ciliary body depending on the eye cornea orientation.

To a lesser extent, the remaining modalities of MNs have been used for the delivery of NPs and MPs. Park *et al.* developed an alternative method for the production of novel types of MNs containing different stacked layers in the needle tip and also MN arrays of complex geometries (112). One of the proposed designs consisted of needle tips made of PLGA (biodegradable polymer) and a needle shaft composed of poly(ethylene glycol) (PEG) containing PLGA MPs encapsulating Vitamin B (riboflavin-5′-phosphate sodium salt dehydrate). This type of MN array was successfully inserted into human cadaver cornea and post-insertion, the PEG layer dissolved leaving the needles embedded in the tissue, creating slow drug release reservoirs. Figure 6c shows a microscopy image of this type of MNs inserted in pig skin. Different layers can be observed clearly, especially the MPs reservoirs in the epidermis. On the other hand, DeMuth *et al.* used PLGA coated MN to deliver plasmid DNA or PLGA NPs into the viable epidermis (116). This research group showed the potential utility of coated MN for the DNA vaccine delivery and the possibility of co-delivery of drug loaded in NPs.

Recently, novel nanoneedle technology has been developed showing potential for delivery of nanomedicines (117). This technology allows interactions with the intracellular environment by bypassing cell membranes facilitating the delivery of drugs and biologics, single-cell stimulation and intracellular sensing (117). Chiappini *et al.* developed a porous silicon nanoneedle system for the cytosolic delivery of CdTe QDs (117). The nanoneedle system was successfully used to delivery QDs to cell cytosol. Furthermore, the same system was tested for the *in vivo* superficial intracellular delivery in a mouse animal model. The obtained results suggest that nanoneedle-mediated delivery is finely-tuned and localized.

### Drug Delivery

MN arrays have been using in combination with NP or MP formulations to deliver many different types of therapeutics across the skin. The main types of MN arrays used for these purposes are solid, hollow and dissolving MNs.

**Fig. 7:**
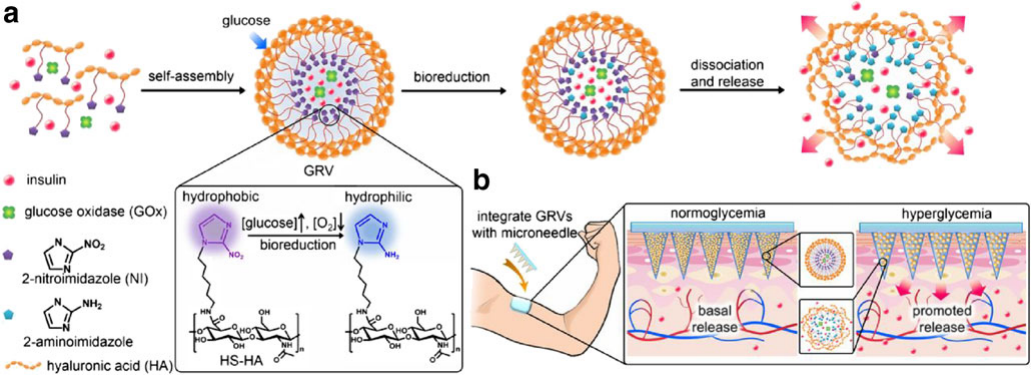
Schematic representation of the formation and triggered insulin delivery of the glucose responsive vesicles (**a**). Schematic of the release from integrated MN/glucose responsive vesicles patch mechanism of action (**b**). Reproduced with permission from (120).

The transdermal delivery of insulin using a combination of MNs and NPs/MPs has been studied by a variety of different research groups (118–120). Ito *et al.* developed chondroitin sulfate dissolving MNs for the delivery of insulin to mice (118). Insulin was incorporated into the chondroitin sulfate matrix adsorbed on two different types of porous MPs (silicon dioxide and calcium silicate). As a control, MNs containing free insulin were also prepared. Both types of MN arrays were employed to administer insulin to mice and 8 h post-application, blood samples were collected. In terms of minimum plasma glucose levels, there were no significant difference across all of the employed approaches. . Nevertheless, after treatment with the MN-MP-insulin arrays, a greater hypoglycaemic effect was recorded, in contrast to that obtained following treatment with MN-insulin arrays. In addition, MN-MP-insulin systems required longer times to reach the minimum glucose level. Recently, Yu *et al.* developed a “*smart insulin patch*” formed by MN arrays containing glucose responsive vesicles loaded with insulin in the tips (120). These novel vesicles are formed by hypoxia-sensitive hyaluronic acid conjugated with a hydrophobic component that can be bio-reduced to hydrophilic under hypoxic conditions (2-nitroimidazole) (Fig. 7a). During enzymatic oxidation of glucose, a local hypoxic microenviroment is generated. In this state the reduction of the hyaluronic acid/2-nitromimidazole conjugate can take place leading to dissociation of the vesicles and the release of insulin (Fig. 7a and b). The system was evaluated in an *in vivo* mouse model of chemically induced type 1 diabetes showing that use of the arrays was able to control glucose levels over the course of several hours. In contrast, Chen *et al.* studied the use of iontophoresis to enhance the transdermal delivery of insulin encapsulated in nanovesicles through holes created in the skin by solid MN arrays (119). Insulin was encapsulated in nanovesicles prepared using soybean lecithin and propylene glycol. After different pressure homogenization cycles or ultrasound methodologies, nanovesicles of different sizes (ranging between 91 and 176 nm), zeta potentials (ranging between −51 and 28 mV) and with insulin entrapment efficiencies of up to 89.05%, were obtained. Iontophoresis alone and combined with MN pre-treatment of the skin allowed higher insulin permeation than the one obtained for passive permeation of the vesicles or free insulin. Among all the different types of nanovesicles that were evaluated, those with positive zeta potential (zeta potential of +27.8 mV and 107.4 nm in diameter) provided the highest insulin permeation. This formulation was then evaluated in a mouse model, in combination with iontophoresis and the reduction in blood glucose levels was shown to be comparable to that obtained after subcutaneous injection of insulin.

**Fig. 8:**
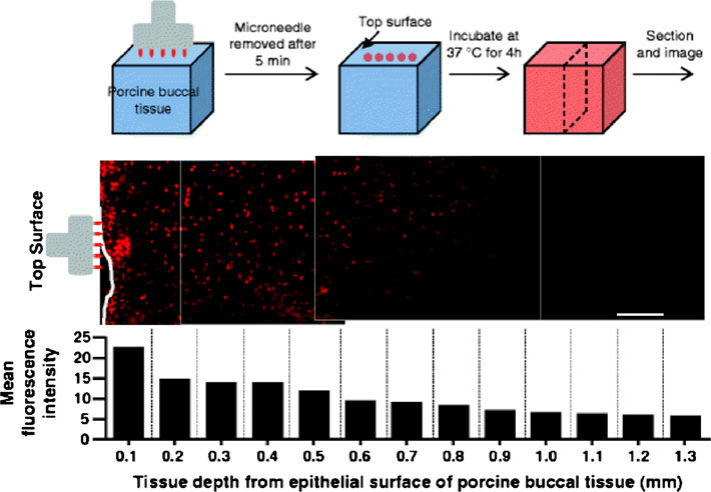
Delivery of doxorubicin in porcine buccal tissue after insertion of MN arrays coated with PLGA NPs and loaded with doxorobucin. Confocal fluorescent microscopy images showing the distribution of the NPs after insertion of the arrays in the porcine buccal tissue. The bar graph shows variation in the mean fluorescence intensity as a function of depth from the insertion site. Scale bar represents: 100 μm. Reproduced with permission from (121).

The treatment of certain cancers, such as those of the oral cavity, where traditional surgical approaches are not always feasible, present interesting challenges. One such novel treatment approach involved the use of doxorubicin loaded PLGA NPs in the treatment of oral cancer (121). In this study, stainless steel MN arrays were coated with the doxorubicin-NP formulation for intratumoral drug delivery in a minimally invasive and painless fashion. The particles employed in this work had an average diameter of 137 nm. MN arrays (700 μm in length and 200 μm in width) coated with the formulation were used to deliver doxorubicin into porcine buccal tissue. Confocal microscopy was used to evaluate the distribution of the NPs showing that the particles could diffuse to a depth of more than 1 mm into the tissue at the insertion point (Fig. 8). In addition to cancer therapeutics, some antioxidants possess anti-carcinogenic properties and therefore can be used for cancer treatment. Paleco *et al.* developed a system to deliver quercentin (antioxidant with anti-inflammatory, anti-carcinogenic and anti-microbial properties) encapsulated in lipidic MPs across the skin (122). In order to enhance the permeation of the lipidic MPs, the skin was pre-treated with silicon MN arrays. The MP formulation was prepared using tristearin as lipidic material and phosphatidylcholine as surfactant. A topical hydrophilic cream was then formulated including the quercetin MPs. This formulation was applied to porcine skin after pre-treatment with silicon MN arrays (36 MNs over 1 cm^2^; MNs of height, 200 μm) showing a significant increase in the permeation profile of quercetin, when compared to the administration of free quercetin. In addition to quercetin, the same research group developed poly(D,L-lactic acid) NPs (sizes 115–150 nm) containing ketoprofen (123). These nanocarriers were capable of sustained and controlled drug release over 7 days when tested *in vitro*. When the particles were applied onto excised porcine skin after pre-treatment with silicon MN arrays, the system was able to release the drug over 24 h.

The use of dyes and pigments as model drugs is a common practice in pharmaceutical science research. These types of molecule allow for rapid detection and function as a proof of concept molecule before beginning to explore the release of therapeutic drugs. Donnelly *et al.* studied the use of dissolving MN arrays as platforms to deliver PLGA NPs containing Nile Red, a model lipophilic molecule (124). Nile Red was used as a model of a hydrophobic pre-formed photosensitiser. NP formulation was prepared following a process that combines emulsion and salting out approaches, yielding particles of approximately 150 nm in diameter. The NPs containing Nile Red were mixed with poly(methylvinylether/maleic anhydride) and then cast into a mould to form MN arrays. The resulting arrays were inserted into excised pig skin and the Nile Red permeation was evaluated using conventional Franz Cell experimentation apparatus. Control patches that did not contain needles were also employed as controls. The permeation results demonstrated that NP permeation was only possible when the NP formulation was applied using dissolving MN arrays. Alternatively, using the same type of NPs and the same type of MN arrays, Gomaa *et al.* showed the ability of this platform to deliver a different type of dye, Rhodamine B (125). In a similar way Zhang *et al.* studied the permeation of PLGA NPs (ca. 160 nm in diameter) in hairless mouse skin pre-treated with solid MNs. In this instance NPs were loaded with coumarin-6 and R-phycoerythrin (a fluorescent probe) and the particles diffused through the created microconduits but were not detected in the receptor compartment. Beyond this, Ke *et al.* developed a MN dissolving system containing PLGA MPs loaded with two dyes (Alexa 488 and Cyanine 5) as model drugs (126). The peculiarity of these MPs was that they were capable of pH triggered release. They included NaHCO_3_ and in contact with acidic pH can stimulate the production of CO_2_ bubbles generating pores in the PLGA structure. The presence of this porous in the particle matrix enhanced the release of the dyes molecules. In this study, the resulting poly(vinylpyrrolidone) (PVP) MN arrays were tested in an *in vivo* rat model and determined that this delivery platform was capable of co-delivering two compounds. In further similar studies, Lee *et al.* used Nile Red encapsulated in a nanostructured lipid carrier (ca. 270 nm in diameter) loaded inside dissolving MN arrays (127). The lipid nanocarriers were prepared by pressure homogenization and were mixed with hyaluronic acid to form the MN arrays following a drawing lithography process. The system was used in a Franz Cell diffusion experiment using dorsal skin from a minipig, thus elucidating the ability of the system to enhance the permeation of Nile Red to the upper layer of the skin.

In addition to these previously described categories of NPs and MPs, other interesting vehicles in NM are QD. The use of QD in combination with MNs have been studied as a promising drug release system. Justin *et al.* developed a formulation containing graphene QD (50–55 nm in diameter and ca. 1.5 nm in height) loaded with lidocaine hydrochloride (128). The obtained QD were used to prepare a composite in combination with chitosan that was used to prepare MN arrays. These arrays showed good mechanical properties to be inserted manually inside chicken skin. Additionally the arrays were able to release *in vitro* between 50 and 70% of the total amount of lidocaine loaded. Additionally, the ability to deliver different molecules from this type of arrays was tested using BSA in combination with iontophoresis. The prepared MN arrays showed promising results. Nevertheless the release test was performed placing the array in contact with the release medium rather than using excised skin or a Franz Cell setup.

### Vaccine Delivery

**Fig. 9:**
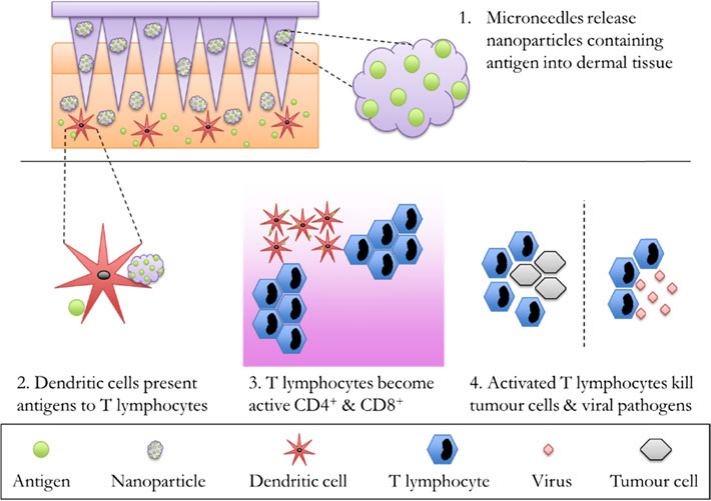
Schematic representation of microneedle arrays penetrating the skin layers releasing nanoparticles, containing vaccine antigens. Antigens are processed by dendritic cells and antigen fragments are presented to T lymphocytes. T lymphocytes become active to CD4+ & CD8+ which then help to destroy tumour cells and viral pathogens.

As stated previously, MN arrays have been used in combination with NP formulations to deliver many different types of therapeutics across the skin and these have expanded to include protein antigen and vaccine therapeutics. Succinctly, successful vaccination is achieved through activation of the adaptive immune system and induction of long lasting memory responses. One of the most effective antigen presenting cell types, dendritic cells (DC), can facilitate robust antigen specific adaptive immunity. DCs process antigenic fragments, presenting these to T cells as peptide sequences bound to MHC (class I and II), resulting in the induction of CD4+ and CD8+ cytotoxic T cells. These activated T cells can act to modulate infection within the body (129). Professional antigen presenting cells such as DCs and Langerhans cells are found in vast quantities within the upper layers of the skin surface. MN technologies facilitate the delivery of therapeutic agents, including vaccines, to these layers of the skin, through penetration of the SC layer and deposition of the NP formulation into these immune cell rich skin layers (Fig. 9).

In the recent past, the incorporation of vaccines into particle-based systems has been proposed as a novel immunisation strategy for the successful delivery of vaccine therapeutics (130). The use of particle-based vaccination systems can aid in the stabilisation of vaccine antigens *in vivo*, in addition to providing controlled and sustained release of the same at the administration site. NPs have been shown, by a variety of different research groups, to display inherent immunogenic properties. To elaborate, it has been shown that induction of T cell immune responses against encapsulated antigens can be achieved using NP technology, with the NPs employed in the studies themselves exhibiting immunogenic properties, comparable to those of traditional adjuvants such as aluminium hydroxide and Freund’s complete adjuvant (131,132). Using NP technology, antigenic material can be adsorbed or conjugated onto the surface of the particles, or directly incorporated within the polymeric matrix (133). Further advantages of the use of antigen/NP formulations include the fact that such formulations protect labile antigens from proteolysis, prolong the uptake of antigenic material by APCs and reduce the release of antigen into systemic circulation (134).

With reference to the use of NP formulations specifically in conjunction with MN devices, Bal *et al.* demonstrated the effective delivery and immunogenicity of N-trimethyl chitosan (TMC) adjuvanted diphtheria toxoid (DT) in 2010 [7]. These DT-loaded TMC NPs were coated onto solid metallic MN arrays, ultimately facilitating their delivery to the skin layers. TMC has a permanent positive charge and is therefore soluble in water over a wide pH range allowed the researchers to efficiently create DT loaded NPs. This paper served as an interesting early example of NP, vaccine and MN combinatorial approaches, suggesting that NPs could act as a depot for vaccine antigens which could be delivered across the skin using a novel MN device (135).

Ovalbumin (OVA) is a widely utilised model antigen in vaccine delivery studies. In one 2011 study, liquid formulations of OVA or OVA conjugated NPs were applied to the skin surface in conjunction with roller microneedling devices, serving as a means of enhancing transdermal delivery. Again, TMC NPs were employed in this mouse study. In this study, significantly higher anti-OVA IgG titres were recorded in those mice which had been treated with OVA conjugated NPs, in comparison to those which had been treated with free OVA solution (136). The authors conceded that this demonstrated that incorporation of protein antigens into NPs can serve to increase their immunogenicity.

It is worth acknowledging however that the formulation of any protein antigen into any NP system will not necessarily result in an immunological response such as those documented previously. Many factors including physical, chemical, and immunological properties of both antigen and NP may be responsible for the ultimate success or failure of a particular formulation. It has been suggested that the optimal vaccine formulation differs for each administration site, with diffusion of antigen into the skin potentially the most important rate-limiting step (135,136).

Although protein based antigens have been the mainstays of vaccination methodologies for many years, novel DNA vaccines have and continue to garner significant attention in the scientific literature. To this end, expanding the remit of NP formulations to incorporate DNA vaccines for transdermal delivery is a logical next step forward in the field. For example, Kumar *et al.*, presented work on plasmid DNA coated cationic PLGA NPs. Once again, a solid MN roller device was used in conjunction the NP formulation but the NPs were coated with plasmid DNA. This unique means of DNA vaccine delivery resulted in the successful immunisation of mice with a gene encoding a protective antigen against anthrax. An interesting outcome of this study was the fact that it highlighted how positively charged plasmid DNA coated NPs elicited a stronger immune response in mice than negatively charged plasmid DNA coated NPs or plasmid DNA alone (137). In contrast to studies using NP decorated with plasmid DNA, DNA can also be nanoencapsulated into the NPs. In one such recent study, McCaffrey *et al.*, illustrated the inherent potential of a nanoencapsulated plasmid DNA vaccine to generate detectable levels of reporter gene expression *in vivo,* when delivered from a novel dissolving MN platform (138). This study was particularly innovative as the NPs were composed of a self-assembling amphipathic peptide, RALA (139,140), which facilitates the intracellular delivery of the DNA across the cell membrane, aids endosomal escape of the DNA cargo and promotes nuclear localisation of the DNA for transcription (138–140). This work perfectly exemplifies how the combination of two such delivery platforms can yield the development of a truly state of the art technology platform for the delivery of, in this instance, nucleic acids*.*

In 2013, Demuth *et al.* employed dissolving MN technologies and NP formulations in a research strategy to study the rapid implantation of controlled-release polymer depots into the cutaneous tissue. A rapidly water soluble polyacrylic acid (PAA) supporting matrix was prepared with needle tips composed of either PLGA NPs or solid PLGA. Upon insertion into skin, the PAA binder rapidly disintegrated, releasing the OVA cargo within 5 min. The PLGA NP continued to release subunit vaccine for a number of weeks (141). Sustained release of vaccine component, localized within the skin layers presents an opportunity for APC targeting and stimulation of long lasting immunity. The outcome of this work was then supported by studies carried out by Zaric *et al.* (129). PGLA NP were suspended in aqueous blends of 20% *w*/*w* PMVE/MA to fabricate dissolving MN arrays, achieving controlled release of OVA antigen into the skin layers. This NP/MN delivery strategy augmented antigen presentation and supported generation of CD8+ cytotoxic T cell responses and CD4+ Th1 immune responses. This study was significant as it not only demonstrated successful prophylactic vaccination against both tumor development and viral challenge, but did so with a single dosing regimen (129). In terms of specific formulation considerations, nanoencapsulation was shown in this same study to improve antigen stability in the MN devices with potential ramifications for the reduction of cold chain storage costs. In the latter study, the central role of skin-resident murine Langerhan’s cells, a sub-set of DCs, in skin immunisation strategies *via* this same NP/MN delivery mechanism, were elucidated (142).

Moving on from the aforementioned studies, ever more complicated NP approaches continue to be developed. For example, Kim *et al.* have studied polyplex DNA vaccines coated onto MN arrays through pH responsive polyelectrolyte multilayer assembly. In brief, solid polycarbonate MN arrays were used to deliver a range of cargoes with robust humoral immune responses demonstrated, in comparison to delivery of the same cargoes *via* conventional SC injection (143). Following this, Hu *et al.* carried out studies to exemplify MN-assisted dendritic cell targeting of NP for transcutaneous DNA immunisation in BALB/c mice (144). The MN-assisted *in vivo* skin penetration of mannosylated grafted cell-penetrating peptide-low molecular weight copolymer NPs was investigated, DC-targeting efficiency was measured and the induction of protective and therapeutic anti-tumour immunity was monitored. In this thorough study, the authors reported that the process efficiently promoted Trp2-specific cellular immune responses, resulting in effective protection against B16 melanoma challenge. The NP formulation strongly induced CD8+ cytotoxic T cells and CD4+ T cells, which secreted interferon-gamma and interleukin 12 cytokines, against melanoma cells. This study, along with others, once again indicates that combinatorial NP/MN strategies have the potential to provide real immunotherapeutic effects.

It has been well documented that MN technologies can assist in the delivery of therapeutic agents to the skin. Therefore, combining this technology with the many positive aspects of NP formulation, specifically in terms of the potential for prolonged antigen stability, depot release and the augmentation of immune responses, serve to make this combinatorial strategy an enticing and exciting area for future research. Initial formulation strategies featuring antigen laden NPs have been published and since then, the field of research has expanded to include the incorporation of NP-based therapeutics into MN devices. Further work on individualized formulation processes for antigen and DNA vaccine components is absolutely required before a vaccine MN product, incorporating NPs, will be realised. However a firm base of literature now exists for future research to build upon and develop.

### Other Uses

MN arrays used in combination with NPs and MPs have been employed in alternative applications, rather than just in drug and vaccine delivery. Their other main field of use is in the delivery of metallic NPs for diagnostic purposes but other uses can also be found in the literature.

Optical coherence tomography (OCT) is a promising diagnostic tool for cancer detection in its earlier stages. The main limitation of this technique however is the low contrast levels in biological tissue, especially between normal and neoplastic tissue. The use of contrast agents such, as gold NPs, can be used to overcome this limitation. For example, Kim *et al.* studied the delivery of gold NPs (71 nm in diameter) across the *SC* and the epithelial barriers after treating the skin with a MN roller device (145). The NP diffusion through the microchannels created by the MN roller was enhanced by the use of ultrasound. The system was successfully used in a model for oral carcinogenesis increasing the contrast level of the OCT technique by approximately 150%. Furthermore, the same research group developed a dissolving MN array containing gold NPs for the same purpose (146). The MN arrays were formulated from sodium carboxymethyl cellulose (CMC) and sucrose. Upon application of the arrays to hamster skin, they dissolved releasing the gold NPs. In order to enhance the permeation of the particles, ultrasound was again employed. The use of the MN/NP system in the treated tissue noticeably enhanced the optical contrast if the OCT images.

MN technology can be used for theranostic applications when combined with QD. Gittard *et al.* used MN arrays made of an acrylate-based polymer to inject QD into porcine skin (147). The developed system was able to inject the QD into the deep epidermis and dermis of the skin. Multiphoton microscopy was then used to track the QD and to visualize the needles inserted in the tissue. Due to the ability to inject QD with minimal pain sensation and without injection site trauma or inflammation, this approach is suitable for further theranostics applications. The same research group also developed organically-modified ceramic MNs for the enhanced delivery of PEG-amine QD solutions into porcine skin (148). The MN arrays were used to create microchannels in the biological tissue and subsequently a solution containing QDs was applied to the treated skin. After the administration of the QD formulation, the nanometric particles were found in the deep epidermal and dermal layers of the porcine skin. The use of MNs has therefore been shown to be a viable alternative for QD delivery through the skin. Nevertheless, more studies should be conducted to provide further evidence for the effectiveness of the QD in theranostic applications after their delivery to the deeper layers of the skin.

NPs and MPs have been used to improve the mechanical properties of MNs. For example, Raja *et al.* developed silk protein MN arrays loaded with silk MPs to increase the mechanical strength of these needles (149). Additionally, the MPs were loaded with BSA and with sulforhodamine and the release of these molecules was evaluated in a 3D collagen gel and in human cadaver skin, respectively. Following the same strategy, Yan *et al.* developed a nanocomposite to prepare MN arrays (150). The presence of 5% (*w*/*w*) of layered double hydroxides NPs inside CMC MNs resulted in improved mechanical properties without compromising the dissolution rate of the MNs in the skin.

**Fig. 10:**
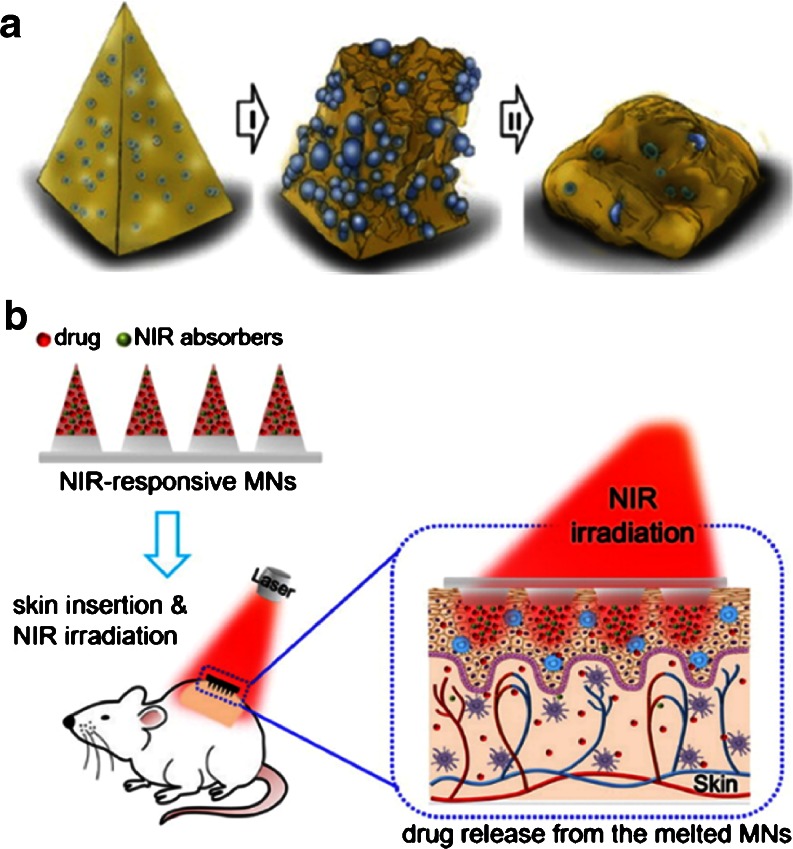
Schematic representation of the mechanism of responsive failure of MN arrays containing hydrogel MPs (**a**). Schematic diagram of triggered transdermal drug delivery using near-infrared light-responsive MN (**b**). Reproduced with permission from (151,152).

Finally, NPs/MPs can be included inside MN arrays to trigger drug release under certain conditions. Kim *et al.* developed biodegradable PLGA MN with internalised hydrogel MPs that enhance drug delivery from the needle tips (151). Hydrogel MPs (10–40 μm) were made by radical cross-linking of N-isopropylacrylamide. Once the MN arrays were inserted into the skin, the particles began to swell and subsequently enabled the failure of the needles (Fig. 10a). This system can be used to deliver both hydrophilic and hydrophobic compounds. Some NP variants harbour interesting radiation responsive properties that have been used to design infrared triggered drug release MN arrays. Chen *et al.* developed polycaprolactone MN arrays containing silica-coated lanthanum hexaboride nanostructures that acted as a local heat source when the array was irradiated with near-infrared radiation (152,153). After irradiation the temperature reached within the arrays was 50°C (152,153), thus inducing a phase transition on the polycaprolactone, leading to the melting of the needles (Fig. 10b). This phenomena triggered drug release from the array. Rhodamine 6G was used as a model molecule to ascertain this behaviour (152,153). Furthermore, the system was tested *in vivo* delivering doxorubicin in a rat model after near-infrared radiation (153).

## CONCLUSIONS

The combination of nanomedicine and MN technology strategies has been made possible due to the exponential growth and development of new (scientific/manufacturing/fabrication) technologies over the course of the last decade. This review has explored and focused on some of the (key) studies charting the concerted development of nanomedicine and MN technology, in a bid to provide a coherent and up-to-date revision of this niche scientific area. The majority of the research works explored in this review have focused on the permeation of NP or on the release of model molecules such as dyes. The main aim of these papers is to demonstrate the feasibility of this technology to deliver NP into the viable skin. Special attention was also paid to the emerging field of protein/DNA based vaccine delivery from NP/MN platforms. As with so many aspects of science and technological developments, it is abundantly clear that although the realm of nanomedicine and MN technologies holds tremendous promise, further research is required in order to fully understand and then exploit its inherent capabilities.

## ABBREVIATIONS

API: Active pharmaceutical ingredient
BSA: Bovine serum albumin
CMC: Carboxymethyl cellulose
MN: Microneedle
MP: Microparticle
NLC: Nanostructured lipid carriers
NM: Nanomedicines
NP: Nanoparticle
OCT: Optical coherence tomography
PEG: Poly(ethylene glycol)
PLA: Poly(lactic acid)
PLGA: Poly(d,l-lactic-co-glycolic acid)
PVP: Poly(vinylpyrrolidone)
QD: Quantum dots
SC: *Stratum Corneum*
SEM: Scanning electron microscopy
SLN: Solid lipid nanoparticles
